# The Duties and Responsibilities of the Editor

**Published:** 1900-06

**Authors:** 


### Editorial.

### THE DUTIES AND RESPONSIBILITIES OF THE EDITOR.

There is no position, possibly, that calls for a greater amount of judicial care outside of the courts than that of an editor, whether that be as head of a daily paper or the conductor of a professional journal. Each, it its way, represents a variety of thought, and to harmonize this contrariety of opinion is an impossibility, and all that can be expected as a result of effort will be to lead this thought, if possible, into channels not subversive to the general good or of professional inharmony.

The ideal editor is one who can divest himself of personal prejudice and view all sides of a controversy without partisan bias. This very exalted condition may not be possible of attainment by any, but the nearer it can be approached will the individual effort be for the general or professional good. This does not mean that an editor must occupy a negative position, one always on both sides of every question. Such a position is always indicative of weakness, and this will be reflected upon the paper or periodical, and in the end it must fail to exert any influence upon its readers.

Independence of thought and a courage to express that thought must be part of the mental training of every editor. He will be met on all sides with opposition, and rarely will he receive the credit due his exertions, but, whether in sunshine or storm, he is forced to maintain that equable temper that regards the one with the same complacency as the other, each the result of natural law and to be continually repeated in his experience.

The independent thinker is necessarily an iconoclast, the breaker of images. This means criticism from those who fail to see the end from the beginning, or who fail to take a broad view of conflicting interests, or who from selfish motives are unwilling to make any sacrifice for the general weal. The true editor cannot permit either criticism or personal interests to influence his pen.

In a professional journal, the organ, in a practical sense, of a large body of intelligent practitioners, it becomes of vital importance that the conductor should write, not as the mouth-piece of a clique, but as an exponent of the truth as he has been able to discover it. He is equally liable to incorrect conclusions as his critics, but the effort on his part to avoid error is influenced by motives that rarely actuate others not thus situated, and are, therefore, more nearly correct.

A professional journal should be conducted upon broad principles. It should be a forum in which could be heard all variety of opinion. Personal clashing should have no place there, but when personal antagonisms upon subjects of general interest are aroused, the views pro and con should find a place upon its pages. Any other course would be subversive of the highest interest of the profession it, in a measure, represents.

The views here outlined have been forced upon the writer's attention by recent occurrences in the dental ranks, and through which the entire attitude and responsibility of the editor to the profession he represents has been forcibly brought into view and, doubtless, has claimed the attention of readers of dental journals.

In the April number of this journal a communication was accepted, after a free use of the editor's privilege of cutting out objectionable matter. It was an answer to the charges made in a previous issue of the *Dental Digest.* These were a severe arraignment of certain parties, members of the Dental Protective Association, because they had accepted the terms of the International Crown Company and paid the demand for royalties upon work done during the life of the patent. This answer, when sent to this journal, was accompanied by the statement that it had been sent to the editor of the *Dental Digest* and had been refused insertion. In the interest of a fair hearing to all sides of a controversy, it was accepted with the changes alluded to previously. It subsequently appeared that the same communication had been sent to other journals. Had this been stated at the time, it would not have appeared, for the reason that the INTERNATIONAL DENTAL JOURNAL cannot be forced into a literary partnership for the dissemination of personal antagonisms.

In connection with this communication was given the decision of Judge Townsend, for the reason that it had not been published previously in full, and it was deemed important that the readers of this journal should have the entire text as delivered. In addition to this the writer felt that the subject required some leading thoughts in the hope that the editor of the *Dental Digest* and president of the Dental Protective Association would frankly explain some very peculiar statements in Judge Townsend's decision. It was thought this was due the members of the Protective Association. It was distinctly understood, however, that the dental journals should not be the medium for a retrial of this case.

The profession received its answer in the April number of the *Dental Digest,* in which the president of the Dental Protective Association publishes in full the aforesaid communication of Dr. Holbrook, and then proceeds to denounce it and those who published it in no measured terms. This editorial seems to the writer to fail in proportion to the amount of temper exhibited. Personal lashings will not satisfy the critical mind,—in fact, they tend to arouse other thoughts not conducive to that harmony and co-operation so essential at this important period.

The position of the editor of this journal has, it is presumed, always been so plain to its readers that it scarcely seems necessary that it should be restated. He has labored earnestly to establish a journal free from all entangling alliances, and to offer a field where all sides were free to enter and be heard with proper limitations. Criticism has been ever welcomed when free from personal abuse. Men wronged by adverse public criticism could find upon its pages room to correct aspersions upon professional character. In a word, it has under its present management been independent of all cliques and ever an open place for all to secure a hearing. The INTERNATIONAL DENTAL JOURNAL has stood for this from the beginning, and should it fall from this high position it must be under other editorial management.

Its attitude towards the Dental Protective Association has never been of a negative character. After the Niagara meeting, where Judge Townsend's decision was explained, this journal endeavored to rouse the apathetic to a full realization of the peril to which those not members of the Protective Association were subjecting themselves by delay in joining that body. That this appeal was productive of good results is well known to the writer. There has never been an occasion since to change the sentiments then expressed, and the publications alluded to were intended, in part, as an incentive to draw out the President of the Protective Association into some effective answer that would silence the fears of the timid in that body. There are great interests at stake in this matter, and nothing is so productive of suspicion as a want of frankness. The President of the Dental Protective Association has been given the confidence of the dental profession as no man previously to his advent, and it is doubtful whether any other man could have held this during all the years he has untiringly worked. It is because of this confidence that his best friends are not willing to have him maligned, as they conceive, without just cause, and they were anxious and had a right to expect something more than a general defamation of his antagonists by name.

The writer has been a member of the Dental Protective Association from the beginning and proposes to continue to the end. He knows that the affairs of this Association have been honestly conducted, and all practitioners of dentistry in this country know of the self-sacrificing energy of its president. That he will lead the good ship into quieter waters is well assured, but he must remember that he occupies a public position and can never be free from the criticisms and false statements of the narrow-minded and the selfish.

This journal has never had any sympathy with those who truckled to the demands of the Crown Company. It will be time enough to do this when the court of last resort has been reached and the decision given, but until this time arrives the entire confidence and earnest co-operation should be accorded to the President of the Dental Protective Association.

